# Gut Microbiome Dysregulation Across Schizophrenia Spectrum Disorders: Bacteria-, Fungi- and Virome-Level Alterations with Molecular and Immunological Implications

**DOI:** 10.3390/ijms27083372

**Published:** 2026-04-09

**Authors:** Răzvan-Ioan Papacocea, Floris Petru Iliuță, Ioana Raluca Papacocea

**Affiliations:** 1 Department of Psychiatry, Faculty of Medicine, Carol Davila University of Medicine and Pharmacy, 020021 Bucharest, Romania; 2 “Dr. Carol Davila” Central Military Emergency University Hospital, 010825 Bucharest, Romania; 3 Department of Psychiatry and Psychology, Faculty of Stomatology, Carol Davila University of Medicine and Pharmacy, 020021 Bucharest, Romania; floris.iliuta@umfcd.ro; 4 Discipline of Medical Physiology, Faculty of Medicine, Carol Davila University of Medicine and Pharmacy, 020021 Bucharest, Romania; raluca.papacocea@umfcd.ro

**Keywords:** schizophrenia spectrum disorders, gut microbiome, short-chain fatty acids, neuroinflammation, microbiome–gut–brain axis, kynurenine pathway, dysbiosis, fecal microbiota transplantation

## Abstract

Schizophrenia spectrum disorders (SSD) are severe psychiatric conditions characterized by disturbances in cognition, emotion, and behavior, with increasing evidence suggesting an involvement of the gut microbiome in their pathophysiology. This PRISMA-informed structured review synthesizes 114 studies using a taxa-centered framework that maps microbial changes across SSD stages and phenotypes and serves as a structural basis for identifying cross-study patterns. Across heterogeneous cohorts, convergent alterations include depletion of short-chain fatty acid (SCFA)-producing taxa (including *Faecalibacterium*, *Roseburia*, and *Coprococcus*) and enrichment of potentially pro-inflammatory and fermentative taxa (such as *Proteobacteria*, *Enterobacteriaceae*, *Streptococcus*, *Collinsella*, and *Desulfovibrio*). These taxonomic patterns suggest potential functional alterations, including reduced SCFA availability. Reduced abundance of butyrate-producing taxa has been associated with impaired intestinal barrier function and increased microbial translocation (e.g., lipopolysaccharide), which may contribute to the activation of immune pathways, including Toll-like receptor 4 signaling and elevated inflammatory markers such as IL-6 and TNF-α. Additional alterations reported across studies include changes in lactate metabolism, bile acid profiles, aromatic amino acid metabolism, and the tryptophan-kynurenine pathway. These pathways may interact with neurobiological processes relevant to SSD, including glutamate-GABA balance, NMDA receptor function, microglial activation, and synaptic regulation, although much of the current evidence remains associative. Multi-kingdom studies and fecal microbiota transplantation models provide further support for the functional relevance of these observations, though causal relationships remain to be fully established. Overall, SSD-associated dysbiosis appears to reflect ecosystem-level metabolic alterations rather than isolated taxonomic abnormalities, supporting a Microbiota–Gut–Immune–Glia conceptual framework and highlighting the gut ecosystem as a potential therapeutic target.

## 1. Introduction

Schizophrenia spectrum disorders (SSD) represent severe and heterogeneous psychiatric disorders that affect perception, cognition, emotion, and behavior. These disorders have been attributed to the interplay of genetic, environmental, and neurodevelopmental factors. Although the pathobiology of SSD remains poorly understood, the disorders have been associated with difficulties in the diagnosis, prognostication, and stratification of treatment [1].

In recent years, the gut–brain axis has been recognized as a relevant biological interface for psychiatric disorders, including SSD. Recent evidence points to the gut microbiome as being associated with immune system, metabolic homeostasis, and neural functions, all of which play a critical role in the pathophysiology of the disorder. There has been a significant accumulation of evidence to suggest consistent differences exist between the gut microbiome composition of individuals suffering from a SSD and healthy controls [2]. This suggests that microbiome alterations may represent a recurring feature of the disorder, although their directionality and causal role remain to be established. Bacteria form the mainstay of the current evidence base. However, emerging data suggest that other microbiome components, including fungi, viruses, and ectopic oral microbes, may interact with bacterial communities and influence overall microbial function, rather than acting as independent drivers [3]. However, it has been noted that the changes to the microbiome associated with the disorder appear to converge toward a limited number of biological processes associated with the immune system, metabolic homeostasis, and brain functions; nevertheless, the specific pathways have not yet been consistently linked to specific microbial taxa or phenotypes of the schizophrenia spectrum [4].

The current review seeks to offer an integrative synthesis of the alterations in the gut microbiome in SSD, based on a taxa-focused approach as the structural framework. This review seeks to offer clarity on the biological mechanisms underlying the alterations in the microbiome in SSD, based on the evidence on the microorganisms implicated in the disorder, as well as the molecular, metabolic, and immunologic studies on the disorder. Information relating to fungal, viral, and oral microbiomes is selectively included where it informs higher-order regulatory or mechanistic processes, rather than being presented as exhaustive or parallel taxonomic findings.

## 2. Results

The results are structured according to a taxa-centered approach, which combines the compositional changes in the microbiota with the molecular, metabolic, and immunological changes that have been associated with them in the literature. Bacteria are the main taxonomic units analyzed, but changes in fungi, viruses, and the oral microbiota have also been included, where available, and combined in Supplementary Table S1 with the bacterial results. Both increases and decreases in microbial taxa have been reported across SSD and their clinical stages. To provide a more meaningful interpretation of the results, the changes in the microbiota have been grouped according to the functional pathways with which they have been associated, including short-chain fatty acid production, fermentation and lactate metabolism, amino acid metabolism, and the tryptophan/kynurenine and immune pathways.

### 2.1. Global Patterns of Gut Microbial Alterations in Schizophrenia Spectrum Disorders

Across the included studies, gut microbiome alterations in SSD were characterized by recurrent yet heterogeneous taxonomic shifts, predominantly involving bacterial taxa (Supplementary Table S1). Despite variability in cohorts, sequencing methodologies and clinical stages, a convergent pattern appears to emerge at the level of functional microbial groups rather than isolated taxa.

One of the most robust findings across independent cohorts was the depletion of SCFA producing genera, including *Faecalibacterium* [5,6], *Anaerostipes, Roseburia* [5,7,8], *Coprococcus* [9,10], *Fusicatenibacter* [11], *Eubacterium* [12,13] and *Blautia* [14,15], which are among the most frequently reported decreases across schizophrenia and related spectrum phenotypes (Supplementary Table S1). Notably, *Faecalibacterium*, the principal butyrate-producing genus [16,17], is consistently reduced, suggesting decreased anti-inflammatory SCFA availability as a recurring ecological feature. Additional metabolic disturbances have been associated with alterations in *Clostridium* species involved in aromatic amino acid metabolism and abnormal phenylalanine derivatives [18,19], as well as by increased representation of *Actinobacteria* [20,21], a phylum frequently described as pro-inflammatory in SSD cohorts [22].

In contrast, several studies report increased abundance of fermentative, lactate-producing and immune-interacting taxa [23,24], including *Prevotella* [25,26], *Megasphaera* [27], *Fusobacterium* [25,28], *Hungatella* [29,30] and *Methanobrevibacter* [29,31] (Supplementary Table S1). *Methanobrevibacter*, an intestinal archaeon capable of interacting with dendritic cells, showed positive correlations with IL-6 and RANTES, suggesting a potential association with immune activation. Similarly, the family *Prevotellaceae* [32] was linked to CD40-mediated systemic immune signaling, and *Ruminiclostridium* [33] was associated with elevated CD40, IL-6 and IL-17 levels, further suggesting the involvement of inflammatory pathway engagement.

Notably, *Lactobacillus* displayed divergent abundance patterns across studies, with both increases and decreases reported depending on population characteristics, disease stage and analytical approach. Despite its conventional classification as a probiotic genus, *Lactobacillus* has frequently been reported as increased [29,34] in schizophrenia cohorts. Its metabolic profile, including lactate production and involvement in glutamate and GABA metabolism, suggests that its role in SSD may depend on the broader ecological context rather than intrinsic probiotic properties.

Diversity analyses also suggest global restructuring of the gut microbiome in SSD. Although α-diversity findings have been heterogeneous across individual studies, recent meta-analytic evidence indicates an overall reduction, particularly in first-episode psychosis [6]. Alterations in β-diversity appear more consistent [35], with repeated evidence of separation between SSD patients and healthy controls [36,37], including in early clinical and high-risk states. While methodological variability warrants cautious interpretation, these findings are consistent with ecosystem-level reorganization rather than isolated taxon-specific abnormalities.

Beyond classical gut-associated bacteria, multiple studies reported enrichment of oral-derived taxa within the intestinal microbiome, suggesting possible oral-to-gut microbial translocation in SSD. Increased gut abundance of genera typically associated with the oral cavity, including *Actinomyces*, *Leptotrichia*, and *Streptococcus parasanguinis* [36,38], has been observed across cohorts. Elevated *Streptococcus gallolyticus*, *Streptococcus gordonii* and *Streptococcus vestibularis* [16] were further associated with increased homocysteine levels and cognitive vulnerability, while transplantation of *Streptococcus vestibularis* into mice induced behavioral alterations [39]. The presence of these immunogenic and metabolically specialized oral taxa may reflect reduced colonization resistance and impaired intestinal ecological stability, mirroring patterns observed in chronic inflammatory conditions.

Although most reported alterations involved bacterial taxa, non-bacterial components of the gut ecosystem were also implicated. Increased abundance of *Candida albicans* [40,41] has been associated with reduced cognitive function and inflammatory activation, potentially involving β-glucan-induced Dectin-1 signaling and downstream NF-κB activation [42]. The fungal genus *Chaetomium* has been reported as increased in first-episode psychosis, suggesting potential involvement in early dysbiosis states [43].

Virome analyses, though limited, identified altered bacteriophage populations, including enrichment of *Carjivirus hominis* and *Mycobacterium virus Renaud18* [16]. Rather than exerting direct neurotropic effects, these viral components may modulate bacterial community structure and metabolic output, indirectly influencing inflammatory and metabolic pathways.

Collectively, the data summarized in Supplementary Table S1 suggest that SSD are associated with reproducible patterns of microbial community reorganization characterized by the depletion of SCFA-producing bacteria, the enrichment of fermentative and immune-interacting taxa, oral microbial translocation, and secondary alterations in fungal and viral communities. These ecological changes provide a conceptual framework for the metabolic, immunological and neurobiological mechanisms detailed in subsequent sections.

### 2.2. SCFA Depletion as a Central Metabolic Feature

One of the most consistently reduced SCFAs appears to be butyrate [44,45]. This alteration is consistent with the depletion of taxa known to produce butyrate such as *Eubacterium* and *Blautia*, suggesting a convergent metabolic signature despite taxonomic heterogeneity. Butyrate maintains intestinal homeostasis by serving as the primary energy substrate for colonocytes, reinforcing tight junction integrity and exerting anti-inflammatory effects through histone deacetylase (HDAC) inhibition. Notably, *Faecalibacterium*, a major inhibitor of inflammation through HDAC inhibition, was consistently reduced across SSD cohorts [46,47]. Additional disruptions in SCFA-related taxa further reinforce this metabolic shift. Members of the Lachnospiraceae family, including Lachnospiraceae UCG-010, displayed stage- and symptom-dependent alterations, being increased in schizophrenia overall but reduced in deficit subtypes [48]. Certain *Clostridium* species, while capable of producing propionate, butyrate and acetate, were also associated with abnormal aromatic amino acid metabolism and inflammatory signatures [18], suggesting functional dysregulation rather than simple SCFA compensation. Increased representation of *Actinobacteria* [49], repeatedly characterized as pro-inflammatory, has also been associated with a shift away from SCFA-mediated immune regulation toward inflammatory activation.

Reduced abundance of butyrate-producing taxa has been associated with increased intestinal permeability and may facilitate the translocation of microbial products such as lipopolysaccharides (LPS) into systemic circulation [50,51]. Consistent with barrier disruption, alterations in mucin-degrading taxa such as *Akkermansia muciniphila* [52] were observed in a stage-dependent manner, being reduced in early SSD and variably altered in chronic cohorts, suggesting dynamic changes in mucosal integrity. Reduced SCFA availability in SSD is associated with impaired mitochondrial function, decreased ATP production, increased reactive oxygen species (ROS) generation and altered mitochondrial gene expression [53]. Taken together, these findings suggest a convergent disruption of SCFA-related metabolic and immune processes in SSD, potentially linking microbial alterations with barrier function, inflammation, and mitochondrial changes.

### 2.3. Barrier Dysfunction, LPS-TLR4 Signaling and Immune Amplification

Increased intestinal permeability may allow circulating LPS to activate Toll-like receptor 4 (TLR4) expressed on peripheral immune cells [51], endothelial cells and microglia. Activation of the TLR4-MyD88-NF-κB signaling cascade is known to induce transcription of proinflammatory cytokines, including IL-1β, IL-6 and TNF-α [54].

Patients with SSD consistently exhibit elevated circulating cytokines and chemokines [55], supporting the presence of sustained low-grade systemic inflammation. Impaired induction of regulatory T cells and reduced interleukin-10 [56] signaling may further contribute to immune imbalance. Correlations between specific microbial taxa and cytokine levels suggest that microbiota-related immune activation could contribute to clinical heterogeneity.

Chronic inflammatory signaling also promotes the activation of indoleamine 2,3-dioxygenase 1 (IDO1), linking immune activation to alterations in neurotransmitter metabolism [57]. In parallel, microbial antigens may induce molecular mimicry mechanisms, generating cross-reactive antibodies against neuronal targets and potentially amplifying synaptic dysfunction in susceptible individuals [58].

Several taxa identified in SSD cohorts are consistent with this immune-activating profile. Members of the phylum Actinobacteria and *Collinsella aerofaciens* were repeatedly characterized as pro-inflammatory [59,60]. The family *Prevotellaceae* has been associated with CD40-mediated systemic immune activation and microglial engagement [61]. Similarly, the archaeon *Methanobrevibacter* (capable of interacting with dendritic cells) showed positive correlations with IL-6 and RANTES [29,62], suggesting that non-bacterial components of the microbiota may also contribute to immune processes. In addition, *Eggerthella lenta* was shown to induce intestinal Th17 responses, further suggesting involvement in proinflammatory signaling pathways [63]. Collectively, these observations may reflect a microbiota-associated pro-inflammatory tendency in SSD.

### 2.4. Lactate Excess, Neurotransmitter Imbalance and Metabolic Neurotoxicity

In parallel with SCFA depletion, multiple studies report increased abundance of lactate-producing bacteria, including *Lactobacillus* [64] and *Streptococcus* spp. [65]. Although traditionally considered beneficial, their overrepresentation in SSD appears context-dependent and potentially metabolically maladaptive. Several of these taxa are metabolically linked to lactate production and hydrogen metabolism, suggesting a shift toward microbial profile.

Elevated lactate levels can cross the blood–brain barrier and have been detected in the brains of patients with schizophrenia [66,67,68]. In some cases, ventricular enlargement has also been reported [69]. Excess lactate has been associated with cerebral acidosis, mitochondrial stress and impaired neuronal energy metabolism, potentially compounding energetic deficits initiated by SCFA depletion. Notably, transplantation of *Streptococcus vestibularis* into mice induced behavioral alterations, suggesting functional relevance of lactate-associated taxa in neurobehavioral phenotypes [70].

Microbiota alterations may also influence neurotransmitter balance through the modulation of glutamate and GABA metabolism [71,72]. Several enriched taxa harbor glutamate decarboxylase, enabling the conversion of glutamate to GABA. Similarly, *Eggerthella* species have been implicated in glutamate metabolism linking microbial activity to neuromodulatory and metabolic processes [73]. In addition, *Lactobacillus* participates in GABA metabolism, further supporting the intersection between fermentative pathways and neurotransmitter regulation [74].

Clinically, these molecular alterations have been associated with cognitive impairment and negative symptom severity, supporting the concept of metabolically mediated neurotoxicity rather than isolated taxonomic imbalance [75]. Overall, these findings suggest a shift toward lactate-related microbial and metabolic alterations in SSD. These changes may be linked to altered energy metabolism and neurotransmitter balance, although their causal role remains unclear.

### 2.5. Microbial Metabolic Pathways and Lipid-Immune Crosstalk

Functional profiling across SSD cohorts suggests broad alterations in microbial metabolic pathways, including reduced glycolysis [76], fatty acid biosynthesis [77] and propionate metabolism, alongside increased lipopolysaccharide (LPS) biosynthesis and amino acid metabolism [78]. Downregulation of key rate-limiting enzymes such as phosphofructokinase and acetyl-CoA carboxylase has been associated with microbial metabolic hypofunction and impaired energy buffering capacity [79].

Altered carbohydrate metabolism was further supported by an increased abundance of the glycoside hydrolase family (GH). GH levels were positively associated with *Bacteroides* spp. [80]. This suggests functional shifts in carbohydrate degradation during disease progression or treatment exposure.

In schizophrenia, an imbalance between primary and secondary bile acids (BAs) has been reported. Patients show increased levels of secondary BAs, particularly deoxycholic acid (DCA) and lithocholic acid (LCA). An altered cholic acid/chenodeoxycholic acid (CA/CDCA) ratio is also observed and is linked to intestinal dysbiosis [81]. Excess secondary BAs, especially the hydrophobic species (LCA and DCA), are known to activate Toll-like receptor 4 (TLR4). This activation can stimulate nuclear factor kappa B (NF-κB) signaling and increases the production of pro-inflammatory cytokines such as interleukin-6 (IL-6) and tumor necrosis factor-alpha (TNF-α) [81]. At the same time, these cytotoxic BAs have been associated with disruption of intestinal tight junctions potentially contributing to increased intestinal permeability and metabolic endotoxemia [82].

Together, these findings suggest a potential mechanistic framework linking dysbiosis → aberrant BA conversion → accumulation of hydrophobic secondary BAs → increased intestinal permeability → elevated LPS → neuroinflammation → cognitive impairment and SSD. Moreover, deficiency of polyunsaturated fatty acids (PUFAs) plays a role in schizophrenia [83].

Certain *Clostridium* species, involved in aromatic amino acid metabolism and phenylalanine derivatives [84], further illustrate how microbial amino acid processing may intersect with dopaminergic and inflammatory pathways [85].

Moreover, metabolite profiles associate with circulating cytokine levels, positioning microbial metabolites as potential mediators between dysbiosis and immune activation in SSD. These findings are consistent with a model in which metabolic reprogramming of the microbial ecosystem may precede and influence host inflammatory responses [86].

### 2.6. Evidence from FMT and Animal Models

Experimental support for microbiota involvement in SSD derives primarily from FMT studies. Transplantation of microbiota from patients with schizophrenia into recipient mice has been shown to induce behavioral phenotypes partially resembling core features of the disorder, including hyperactivity, anxiety-like behavior and reduced sociability [87]. These behavioral changes are accompanied by distinct brain transcriptomic alterations not observed in control-transplanted animals [33,88,89].

Beyond whole-community transplantation, targeted experiments have demonstrated that the administration of *Streptococcus vestibularis* (a taxon enriched in SSD) can induce behavioral alterations in recipient mice, including hyperactivity and social deficits [70,90]. This finding suggests the functional relevance of specific microbial taxa and suggests that certain organisms may exert disproportionate influence within the broader dysbiotic ecosystem.

RNA sequencing analyses in FMT-recipient mice have identified differentially expressed genes enriched in immune-related processes, receptor signaling and synaptic pathways [89,91]. In addition, partial transcriptomic overlap between schizophrenia patient cortex and recipient mouse brain tissue suggests cross-species convergence at the molecular level. Complementary evidence from maternal immune activation models indicates that early-life microbiota remodeling and alterations in tryptophan-kynurenine metabolism may produce behavioral and molecular phenotypes relevant to SSD. Similarly, combined maternal microbiota disturbance and genetic susceptibility (e.g., NOD2 deficiency) have been associated with exacerbated offspring brain developmental alterations and schizophrenia-like behaviors, suggesting that early-life microbial-immune interactions may influence neurodevelopment [57,92]. Human cohort studies also report that the maternal prenatal gut microbiome composition is associated with early neurodevelopment, supporting translational relevance for developmental origins of psychiatric risk [93].

## 3. Discussion

Based on Supplementary Table S1 and the convergent findings from the literature summarized therein, SSD appear to be characterized by reproducible shifts in key bacterial taxa. Among the increased taxa, the most consistently reported are *Proteobacteria*, *Enterobacteriaceae*, *Streptococcus* (including *Streptococcus sobrinus*), *Collinsella*, and *Desulfovibrio*, all associated with pro-inflammatory signaling. Conversely, several beneficial SCFA-producing taxa are reduced. The most consistently decreased bacteria include *Faecalibacterium* (and from these, the most significant were *F. prausnitzii*), *Roseburia*, *Coprococcus*, *Ruminococcaceae*, and *Bifidobacterium*, taxa linked to butyrate production and gut barrier integrity. These taxa represent the most consistently reported microbial patterns across the identified studies and provide context for the variability observed across studies (as summarized in Supplementary Table S1).

Together, this pattern reflects a shift from an anti-inflammatory, SCFA-rich ecosystem toward a pro-inflammatory and metabolically dysregulated microbial profile in SSD. In contrast, several other taxa reported across studies show inconsistent directions of change or are supported by a limited number of studies (Supplementary Table S1), suggesting that these findings remain context-dependent and should be interpreted with caution.

Taking this pattern into account, our study suggests that gut microbiome alterations in SSD are best conceptualized as a multi-functional and metabolic reprogramming of the microbial ecosystem, rather than as reproducible abnormalities of individual taxa. Across heterogeneous cohorts, the most stable signal emerging from the literature is convergence toward a dysbiotic state characterized by reduced SCFA availability [94,95,96,97,98], increased fermentative activity [99,100,101] and sustained immune activation [102,103,104]. Importantly, this apparent convergence should be interpreted in the context of substantial heterogeneity across studies, including differences in sequencing methodologies (e.g., 16S rRNA sequencing versus metagenomic approaches), patient populations, disease stages, dietary background, and comorbidities. Moreover, antipsychotic treatment represents an important confounding factor, as these medications can independently alter gut microbiota composition and metabolic profiles, thereby complicating the interpretation of disease-specific microbial changes observed across schizophrenia cohorts. This effect may contribute to variability across studies and should be considered when interpreting microbiome alterations in medicated populations. These sources of variability likely contribute to inconsistencies in reported microbial patterns and may partially explain why certain taxa exhibit divergent directions of change across cohorts, highlighting the need for cautious cross-study interpretation. Reduced SCFA, particularly butyrate levels, may represent a key mechanistic link in SSD. SCFA deficiency has been associated with impaired tight junction integrity and increased gut permeability. This may facilitate LPS translocation and systemic inflammation, including increased IL-6 and TNF-α. The resulting immune activation may contribute to microglial activation and is predominantly associated with negative and cognitive symptom severity [105]. The gut microbiota may also modulate glial cell activity, as well. It influences microglial polarization toward pro- or anti-inflammatory states and affects astrocyte and oligodendrocyte function. In the context of dysbiosis, increased microglial glutamate release has been reported, along with reduced long-term potentiation (LTP) and structural alterations within the central nervous system. These mechanisms are consistent with the proposed Microbiota–Gut–Immune–Glia (MGIG) axis, which is highly relevant for SSD [106].

Specific microbial taxa were differentially associated with symptom domains. Increased *Streptococcus sobrinus* and members of *Methylophilaceae* have been associated with negative and cognitive symptoms, while *Bacteroides plebeius* was associated with positive symptoms. Network analysis identified a defined microbiota–metabolite–symptom axis. Elevated *Streptococcus sobrinus* was associated with changes in 7-aminomethyl-7-deazaguanine and vitamin D2, which in turn were associated with cognitive impairment. These findings support a functionally interconnected pathway rather than isolated taxon–symptom correlations [107]. From a clinical perspective, microbiota-related findings may hold potential for the development of novel biomarkers and therapeutic strategies in SSD. Microbial signatures could contribute to patient stratification, disease monitoring, or the identification of specific pathophysiological subtypes. In addition, microbiome-targeted interventions, including dietary modulation, probiotics, prebiotics, or fecal microbiota transplantation, have been proposed as potential adjunctive approaches.

Across SSD, recurring microbial patterns have been described. These include increased *Proteobacteria* and *Enterobacteriaceae* and decreased beneficial taxa such as *Firmicutes* spp., *Bifidobacterium*, and *Lachnospira* (Supplementary Table S1). This profile is consistent with a pro-inflammatory state and a loss of SCFA producers. Although the present article does not provide detailed taxon-specific findings for schizophrenia, its results are consistent with this broader biological framework [106].

Gut microbiota dysbiosis may interfere with the glutamate-GABA cycle by altering the availability of precursor amino acids and by modulating enzymes involved in glutamate decarboxylation and glutamine recycling. Excess glutamate has been associated with excitotoxic stress and may contribute to N-methyl-D-aspartate receptor (NMDAR) hypofunction through receptor internalization and redox imbalance. In parallel, quinolinic acid, another kynurenine metabolite, may exert excitotoxic and pro-inflammatory effects [108]. Together, dysregulation of the glutamate-GABA cycle and the kynurenine pathway have been associated with impaired excitatory-inhibitory balance, microglial activation, and synaptic dysfunction. These interconnected mechanisms provide a biologically plausible framework linking gut microbial metabolic alterations to early pathophysiological changes observed in SSD, including first-episode psychosis [109,110]. In line with the described glutamate-GABA imbalance, inflammation-driven diversion of tryptophan toward the kynurenine pathway reduces serotonin synthesis and increases neurotoxic metabolites. Furthermore, IL6 and TNF-α have been implicated in BBB disruption by altering tight junction integrity and increasing endothelial permeability [111]. But neuroinflammation alone is insufficient to explain psychosis, suggesting that additional environmental triggers are required. Chronic stress has been proposed as a contributing factor of latent infections and immune imbalance, further amplifying neuroinflammatory cascades. Contemporary Western lifestyles characterized by chronic stress, low-grade systemic inflammation, and gut dysbiosis are framed as evolutionarily mismatched environments that may increase schizophrenia susceptibility. Some studies have proposed shifting the therapeutic focus beyond symptom suppression toward identifying and targeting microbial infection, inflammation, gut dysbiosis, and chronic stress as modifiable upstream drivers [112]. Studies performed in mice [113] and humans suggest that the same genetic risk could be associated with different microbiota, depending on sex. In humans, females generally exhibit higher alpha diversity and increased abundance of taxa such as *Akkermansia* and *Bifidobacterium*, which are often linked to beneficial metabolic and immune processes. In contrast, males tend to show relatively higher levels of taxa like *Prevotella* and *Escherichia*, patterns that may relate to differences in diet, hormones, and immune responses. These sex-specific microbial signatures suggest that sex hormones and host physiology may influence gut microbiota composition across adulthood. Understanding these differences is important for microbiome research and may have implications for disease risk and personalized interventions [114]. It is important to note that a substantial proportion of functional interpretations in the present review are inferred from taxonomic associations rather than directly measured metabolomic or metagenomic data. While some studies provide direct metabolomic, metagenomic, or experimental evidence, many mechanistic links are based on known metabolic capacities of specific taxa and should therefore be interpreted with caution. Finally, the directionality of the association between gut microbiota alterations and SSD remains unclear. It is difficult to distinguish whether observed microbial changes represent causal contributors to disease pathophysiology, downstream consequences of the disorder, or effects related to lifestyle factors and treatment exposure. Thus, more longitudinal and mechanistic studies are necessary to better clarify causal relationships.

## 4. Materials and Methods

This integrative review was conducted using a structured and systematic search strategy and reported in accordance with the relevant recommendations of the Preferred Reporting Items for Systematic Reviews and Meta-Analyses (PRISMA) 2020 guidelines to ensure transparency in the identification and selection process. However, it was not designed as a formal systematic review and therefore did not include a standardized risk-of-bias assessment or quantitative synthesis.

### 4.1. Eligibility Criteria

#### 4.1.1. Inclusion Criteria

Articles were eligible for inclusion if they met the following criteria: (i) original research articles/reviews reporting gut microbiome alterations in schizophrenia spectrum disorders (including schizophrenia and first-episode psychosis); (ii) studies conducted in human populations, with bacterial taxa as primary microbiome findings; (iii) translational studies directly derived from human schizophrenia-associated microbiome data (e.g., fecal microbiota transplantation from patients with schizophrenia to animal models); (iv) a limited number of animal studies, included only when they provided direct mechanistic insight relevant to schizophrenia-associated microbiome alterations and could not be addressed by available human data; (v) studies reporting the direction of microbial changes (increased or decreased abundance) and/or providing molecular, metabolic, or immunological data related to microbiome alterations; (vi) studies relevant to gut-microbiome molecular mechanisms; and (vii) articles published in English within the last 10 years prior to the final search date.

#### 4.1.2. Exclusion Criteria

The following were excluded: (i) studies without relevance to SSD or without mechanistic relevance to microbiome-related pathways; (ii) studies focusing on psychiatric disorders outside the schizophrenia spectrum; (iii) editorials, conference abstracts, and case reports without original data; (iv) studies lacking clear taxonomic identification or comparative microbiome analysis or gut-microbiome molecular data; and (v) articles published in languages other than English or outside the predefined time frame.

### 4.2. Information Sources and Search Strategy

An integrative search was conducted in the following electronic databases: PubMed, Scopus, and Web of Science, from database inception to December 2025. Search terms combined controlled vocabulary and free-text keywords related to schizophrenia spectrum disorders and the gut microbiome, including but not limited to: “schizophrenia”, “SCFA”, “schizophrenia spectrum”, “gut microbiome”, “SSD”, “inflammation”, “mycobiome”, and “virome”. Search strategies were adapted to the syntax of each database. In addition, reference lists of included articles and relevant reviews were manually screened to identify further eligible studies.

### 4.3. Selection Process

**Figure 1:**
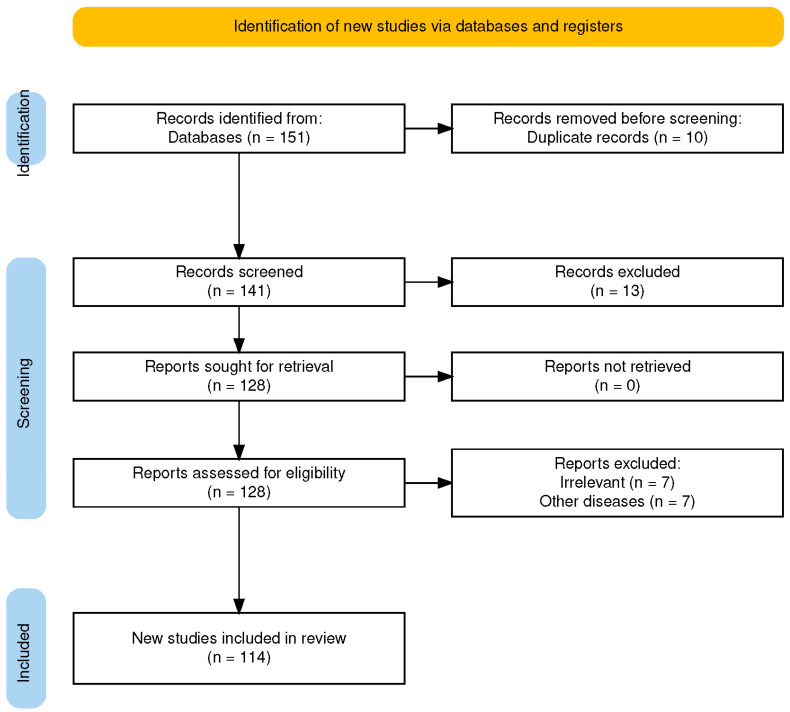
PRISMA 2020 flow diagram. Flow diagram illustrating the identification, screening, eligibility assessment, and inclusion of studies in accordance with PRISMA 2020 guidelines. A total of 114 studies were included in the qualitative synthesis.

All records retrieved from searches were imported into a database and duplicate entries were removed. Titles and abstracts were screened for relevance based on predefined eligibility criteria. Full-text articles were subsequently assessed for inclusion. Studies that did not meet the inclusion criteria were excluded with the reasons documented. The selection process followed PRISMA 2020 recommendations and is summarized in a PRISMA flow diagram (Figure 1).

### 4.4. Data Collection Process

Data were extracted from each included study using a standardized template. Extracted information included: author and year of publication; microbiome sampling type; microbial taxa reported with direction of change; and any reported molecular, metabolic, or immunological findings linked to microbiome alterations. Only explicitly reported data were included; no assumptions were made where information was absent or unclear.

### 4.5. Study Risk of Bias Assesment

A formal risk-of-bias assessment was not performed, as this review aimed to integrate findings across heterogeneous study designs rather than conduct a quantitative comparison. However, study quality and methodological limitations were considered during interpretation and are discussed in the Limitations section.

### 4.6. AI Use Disclosure Statement

The authors would like to transparently acknowledge the use of artificial intelligence tools solely for linguistic refinement and assistance with English phrasing during manuscript preparation. All scientific content, data synthesis, conceptual framework, interpretations, and conclusions are entirely the authors’ own and are based exclusively on the cited literature. No ideas, analyses, or original scientific contributions were generated by artificial intelligence.

## 5. Limitations

Several limitations should be considered when interpreting the findings synthesized in this review. First, most human microbiome studies in SSD are cross-sectional in design. Consequently, causal relationships between gut dysbiosis and disease onset or progression cannot be definitively established. Although FMT studies provide experimental support for microbiota-associated behavioral and molecular alterations, translation from animal models to human pathophysiology remains limited.

Second, substantial methodological heterogeneity exists across studies, including differences in sequencing platforms, bioinformatic pipelines, sampling procedures, dietary control, geographic background and clinical characterization. Most investigations rely on 16S rRNA sequencing, which limits strain-level resolution and constrains direct functional inference. As a result, functional interpretations often depend on predictive tools rather than direct metabolomic measurements.

Third, SSD are biologically and clinically heterogeneous. Variability in inflammatory status, metabolic comorbidities, body mass index, antipsychotic exposure and disease stage may significantly influence microbiome composition. Few studies adequately stratify patients according to these variables, which likely contributes to taxonomic inconsistency across cohorts.

Fourth, confounding factors such as diet, smoking, substance use, and medication effects are difficult to fully control and may independently influence microbial composition and metabolic output. Antipsychotic treatment, in particular, has been shown to affect weight, glucose metabolism and microbiome structure, complicating the interpretation of disease-specific effects.

Finally, while increasing attention is being directed toward fungal and viral components of the microbiome, current evidence remains limited and largely descriptive. The functional role of the mycobiome and virome in SSD requires further mechanistic and longitudinal investigation.

Taken together, these limitations highlight the need for standardized multi-omics approaches, longitudinal study designs and well-stratified patient cohorts to clarify the temporal and mechanistic relationships between gut microbiota alterations and SSD pathophysiology.

## 6. Conclusions

This review provides a taxa-centered and functionally integrated synthesis of gut microbiome alterations across SSD, moving beyond descriptive compositional analyses toward a mechanistic framework. By systematically mapping bacterial, fungal, viral microbiomes within a unified ecological model, the study suggests that SSD-related dysbiosis converges on a limited number of immune–metabolic pathways rather than isolated taxonomic abnormalities.

An important element of this article is the identification of SCFA depletion (particularly butyrate deficiency) as a potential central metabolic feature, linking microbial alterations to barrier dysfunction, LPS translocation, and IL-6/TNF-α–mediated immune amplification. The review further integrates bile acid dysregulation, lactate excess, and aromatic amino acid metabolism into a coherent inflammatory framework. Importantly, the study contributes to the field by functionally connecting dysbiosis to the glutamate–GABA cycle and kynurenine pathway activation, thereby linking microbial metabolism to NMDAR dysfunction, microglial activation, and synaptic instability. This mechanistic convergence provides a biologically plausible bridge between gut ecology and core cognitive and negative symptom domains.

Another strength of this work is the incorporation of multi-kingdom alterations (mycobiome, virome, archaeome into the SSD model, expanding beyond bacteria-only paradigms. By integrating FMT evidence, immune signaling data, and metabolic reprogramming, the article reframes SSD-associated dysbiosis as a systems-level ecological shift. In the frame of the previously mentioned limitations, this work provides an integrative Microbiota–Gut–Immune–Glia conceptual model, highlighting metabolic reprogramming as the core feature of microbiome involvement in SSD, and identifying the gut ecosystem as a potential therapeutic target.

## Data Availability

No new data were created or analyzed in this study. Data sharing is not applicable to this article.

## Supplementary Materials

The following supporting information can be downloaded at: https://www.mdpi.com/article/10.3390/ijms27083372/s1.
