# Supplementary material for: Gut Microbiome Dysregulation Across Schizophrenia Spectrum Disorders: Bacteria-, Fungi- and Virome-Level Alterations with Molecular and Immunological Implications
Source: Int J Mol Sci. 2026 Apr 9;27(8):3372. doi: 10.3390/ijms27083372 (PMC13116486; doi:10.3390/ijms27083372)
Supplement: Supplementary file 1 [file ijms-27-03372-s001.zip › ijms-4205065 Supplementary Update.pdf]

## Supplementary Table S1

**Legend:** ↑ (increased); ↓ (decreased)ITSs SSD (Schizophrenia spectrum disorder), SCZ (Schizophrenia), FEP (First-episode psychosis), SCZ-P (Schizophrenia with Positive Symptoms), SCZ-P (Schizophrenia with Negative Symptoms), SCZ-OW (Overweight patients with schizophrenia), *(originally from mouth, possible translocation in the gut) MGS - shotgun metagenomic sequencing; NA-nor applicable; 16S-16S rRNA sequencing; MR- mendelian randomization; ITSs -Internal Transcribed spacer sequencing; qPCR -quantitative Polymerase Chain reaction;

| BACTERIA | DISORDER | VARIATION | STUDY TYPE | MEDICATION STATUS | METHOD | REFERENCES |
|---|---|---|---|---|---|---|
| **Acidaminococcus** | SCZ | ↑ | Systematic review | NA | NA | [5] |
| **Actinobacteria [class]** | SCZ | ↑ | Case-control study<br>Case-control study<br>Case-control study | Mixed (mainly antipsychotics)<br>NA<br>Mixed | MGS<br>16S + MR<br>16S | [20]<br>[21]<br>[22] |
| **Actinomyces spp.** | SSD<br>SSD | ↑<br>↑ | Case-control study<br>Case-control study | Mixed (mainly antipsychotics)<br>Treated (antipsychotics) | MGS<br>16S | [36]<br>[38] |
| **Agathobaculum butyriciproducens** | FEP | ↓ | Case-control study | Drug naive | Multi-omics | [45] |
| **Aggregatibacter** | Acute Phase | ↓ | Case-control study | Mixed | 16S | [107] |
| **Akkermansia muciniphila** | SSD | ↓ | Case-control study<br>Case-control study | Mixed (mainly antipsychotics)<br>Mixed | MGS<br>MGS | [20]<br>[36] |
| **Alloprevotella*** | SCZ [elders] | ↓ | Case-control study | Treated (antipsychotics) | 16S | [38] |
| **Anaeroglobus genus** | SCZ-P | ↑ | Case-control study | Mixed | 16S | [59] |
| **Anaerostipes** | SCZ | ↓<br>↓ | Systematic review<br>Narrative Review | NA<br>NA | NA<br>NA | [5]<br>[7] |
| **Anaerostipes hadrus** | FEP | ↓ | Case-control study | Drug naive | Multi-omics | [45] |
| **Archaeorhizomyces [fungi]** | FEP | ↑ | Case-control study | Drug naive | ITSs | [51] |
| **Arthrobacter [genus]** | SCZ | ↑ | Case-control study | Mixed | Multi-omics | [15] |
| **Ascomycota** | SCZ | ↓ | Case-control study | Mixed | ITSs | [41] |

| | | | | | | |
|---|---|---|---|---|---|---|
| **Aspergillus [fungi]** | FEP | ↓ | Case-control study | Drug naive | ITSs | [51] |
| **Bacteroides** | FEP<br>SCZ | ↑<br>↓ | Meta-analysis<br>Case-control study | NA<br>Treated | NA<br>16S | [6]<br>[29] |
| **Parabacteroides** | SCZ | ↑ | Case-control study | Treated | MGS | [9] |
| **Bacteroides plebeius** | SSD | ↑ | Systematic review | NA | NA | [40] |
| **Bacteroidetes** | SCZ | ↑ | Case-control study | Mixed | 16S | [18] |
| **Basidiomycota** | SCZ | ↑ | Case-control study | Mixed | ITSs | [41] |
| **Bifidobacterium** | FEP<br>SCZ<br>SCZ | ↓<br>↓<br>↓ | Meta-analysis<br>Case-control study<br>Narrative Review | NA<br>Drug naive<br>NA | NA<br>Multi-omics<br>NA | [6]<br>[45]<br>[12] |
| **Bifidobacterium longum** | SSD | ↑ | Systematic review | NA | NA | [40] |
| **Bilophila wadsworthia** | SCZ | ↑ | Case-control study | Treated | MGS | [9] |

| | | | | | | |
|---|---|---|---|---|---|---|
| **Blautia** | SCZ | ↓<br>↓ | Meta-analysis<br>Case-control study | NA<br>Mixed | NA<br>Multi-omics | [6]<br>[15] |
| **Botrytis cinerea [fungi]** | SCZ | ↑ | Case-control study | Treated | MGS | [16] |
| **Butyricicoccus** | SCZ | ↓<br>↑ | Meta-analysis<br>Cohort Study | NA<br>Treated | NA<br>16S | [6]<br>[26] |
| **Candida Albicans [fungi]** | SSD | ↑<br>↑<br>↑ | Systematic review<br>Cohort Study<br>Case-control Study | NA<br>Mixed<br>NA | NA<br>ITSs<br>qPCR | [40]<br>[41]<br>[42] |
| **Candidatus Soleaferrea** | Deficit-SCZ | ↑ | Case-control study | Mixed | 16S | [25] |
| **Carjivirus hominis [virus]** | SCZ | ↑ | Case-control study | Treated | MGS | [16] |
| **Catenibacterium** | SCZ | ↑ | Case-control study | Mixed | 16S | [67] |
| **Chaetomium [fungi]** | FEP | ↑ | Narrative Review | NA | NA | [43] |
| **Clostridium** | SCZ | ↑<br>↑ | Case-control study<br>Case-control study | Mixed<br>Treated | 16S<br>16S | [18]<br>[19] |
| **Clostridium innocuum group** | SCZ | ↑ | Case-control study | Treated | 16S | [29] |
| **Collinsella [genus]** | SCZ<br>SCZ-P<br>SCZ-OW | ↑<br>↑<br>↑ | Meta-analysis<br>Case-control study<br>Case-control study | NA<br>Mixed<br>Treated | NA<br>16S<br>16S | [6]<br>[59]<br>[60] |
| **Collinsella aerofaciens** | SCZ | ↑ | Case-control study | Treated | MGS | [9] |
| **Coprobacillus** | SCZ | ↑ | Case-control study | Treated | 16S | [29] |

| | | | | | | |
|---|---|---|---|---|---|---|
| **Coprococcus** | SCZ | ↓<br>↓<br>↓ | Systematic review<br>Case-control study<br>Meta-analysis | NA<br>Treated<br>NA | NA<br>MGS<br>NA | [5]<br>[9]<br>[10] |
| **Coriobacteriaceae [familie]** | SCZ-P | ↑ | Case-control study | Mixed | 16S | [59] |
| **Corynebacterium** | SSD | ↑ | Narrative Review | NA | NA | [31] |
| **Cronobacter spp** | SSD | ↑ | Systematic review | NA | NA | [40] |
| **Debaryomycetaceae [fungi]** | FEP<br>SSD | ↓<br>↓ | Narrative Review<br>Case-control study | NA<br>Drug naive | NA<br>ITSs | [43]<br>[51] |
| **Desulfovibrio** | SCZ | ↑ | Meta-analysis | NA | NA | [6] |
| **Dialister [genus]** | SCZ | ↑ | Case-control study | Treated | 16S | [30] |
| **Dorea [genus]** | SCZ-P | ↑ | Case-control study | Mixed | 16S | [59] |
| **Eggerthella** | SCZ | ↑<br>↑ | Meta-analysis<br>Case-control study | NA<br>Treated | NA<br>16S | [6]<br>[29] |
| **Eggerthella lenta** | SSD | ↑ | Case-control study | Mixed (mainly antipsychotics) | MGS | [20] |
| **Eisenbergiella** | SCZ | ↑ | Case-control study | Treated | 16S | [29] |
| **Enterobacteriaceae [family]** | SCZ | ↑<br>↑ | Systematic review<br>Case-control study | NA<br>Mixed | NA<br>16S | [5]<br>[59] |
| **Enterococcaceae** | SCZ | ↑ | Case-control study | Mixed | MGS | [35] |
| **Enterococcus** | SCZ | ↑ | Systematic review | NA | NA | [23] |
| **Epsilonproteobacteria [genus]** | SCZ | ↑ | Case-control study | Treated | 16S | [13] |
| **Erysipelotrichaceae** | SCZ | ↑ | Case-control study | Mixed | MGS | [35] |
| **Eubacterium** | SCZ | ↓<br>↓ | Narrative Review<br>Case-control study | NA<br>Treated | NA<br>16S | [12]<br>[13] |
| **Eubacterium fissicatena group** | SCZ | ↑ | Case-control study | Treated | 16S | [29] |
| **Eubacterium siraeum** | SSD | ↑ | Systematic review | NA | NA | [40] |
| **Eubacterium ventriosum** | SCZ | ↓ | Case-control study | Treated | MGS | [16] |

| | | | | | | |
|---|---|---|---|---|---|---|
| **Euryarchaeota [ archaea phylum]** | SCZ | ↑ | Case-control study | Mixed | 16S | [59] |
| **Faecalibacterium** | SCZ | ↓<br>↓<br>↓ | Systematic review<br>Meta-analysis<br>Systematic Review | NA<br>NA<br>NA | NA<br>NA<br>NA | [5]<br>[6]<br>[46] |
| **Faecalibacterium duncaniae** | SCZ | ↓ | Case-control study | Treated | MGS | [16] |
| **Faecalibacterium prausnitzii** | SCZ | ↓<br>↓ | Case-control study<br>Narrative Review | Treated<br>NA | MGS<br>NA | [9]<br>[17] |
| **Firmicutes** | SCZ | ↑<br>↓ | Case-control study<br>Case-control study | Mixed<br>Mixed | Multi-omics<br>16S | [24]<br>[59] |
| **Flandersviridae** | SCZ | ↑ | Case-control study | Mixed | MGS | [52] |
| **Flavonifractor plautii** | SCZ | ↑ | Case-control study | Treated | MGS | [9] |
| **Flexispira** | Acute Phase | ↓ | Case-control study | Mixed | 16S | [107] |
| **Fusicatenibacter** | SCZ | ↓ | Systematic review | NA<br>Mixed | NA<br>16S | [5]<br>[11] |
| **Fusobacterium** | Deficit-SCZ | ↑ | Case-control study | Mixed | 16S | [25] |
| **Gammaproteobactria [class]** | SCZ | ↑ | Systematic review | NA | NA | [5] |
| **Gemmiger** | SCZ [elders] | ↓ | Case-control study | Mixed | 16S | [11] |
| **Geopora [fungi]** | FEP | ↑ | Case-control study | Drug naive | ITSs | [51] |
| **Gordonibacter [genus]** | SSD | ↓ | Mendelian Randomization | NA | MR | [32] |
| **Haemophilus [genus]** | Acute Phase | ↑ | Case-control study | Mixed | 16S | [27] |
| **Holdemanella** | SCZ | ↑ | Case-control study | Treated | 16S | [30] |
| **Hungatella** | SCZ | ↑<br>↑ | Case-control study | Treated<br>Treated | 16S<br>16S | [29]<br>[30] |
| **Klebsiella [pneumoniae]** | SSD | ↓ | Case-control study | Mixed (mainly antipsychotics) | MGS | [20] |

| | | | | | | |
|---|---|---|---|---|---|---|
| **Lachnoclostridium** | SSD | ↑ | Case-control study | Mixed (mainly antipsychotics) | MGS | [20] |
| **Lachnospira** | SCZ | ↓ | Meta-analysis | NA | NA | [6] |
| **Lachnospiraceae** | SCZ | Context-dependent | Narrative Review | NA | NA | [48] |
| **Lachnospiraceae UCG-004** | Deficit-SCZ | ↓ | Case-control study | NA | NA | [25] |
| **Lachnospiraceae UCG-010** | Deficit-SCZ<br>SCZ | ↓<br>↑ | Case-control study<br>Narrative Review | Mixed<br>NA | 16S<br>NA | [25]<br>[4] |
| **Lactobacillaceae** | SCZ | ↑ | Systematic review | NA | NA | [5] |
| **Lactobacillus** | FEP<br>SCZ | ↓<br>↑<br>↑ | Meta-analysis<br>Case-control study<br>Case-control study | NA<br>Treated<br>Treated | NA<br>16S<br>16S | [6]<br>[29]<br>[34] |
| **Leptotrichia*** | SCZ [elders] | ↑ | Case-control study | Treated | 16S | [38] |
| **Libanicoccus** | Deficit-SCZ | ↑ | Case-control study | Mixed | 16S | [25] |
| **Ligilactobacillus** | SCZ | ↑ | Case-control study | Treated | 16S | [29] |
| **Limosilactobacillus** | Deficit-SCZ | ↑ | Case-control study | Mixed | 16S | [25] |
| **Marvinbryantia [genus]** | SSD | ↑ | Mendelian Randomization | NA | MR | [32] |
| **Megamonas [genus]** | SCZ | ↑ | Case-control study | Treated | 16S | [30] |
| **Megasphaera** | SCZ | ↑<br>↑ | Meta-analysis<br>Case-control study | NA<br>Mixed | NA<br>16S | [6]<br>[18] |

| | | | | | | |
|---|---|---|---|---|---|---|
| **Methanobrevibacter** | SCZ | ↑<br>↑ | Case-control study | Treated<br>NA | 16S<br>NA | [29]<br>[31] |
| **Methylomonas** | Acute Phase | ↓ | Case-control study | Mixed | 16S | [107] |
| **Mimiviridae [virus]** | SCZ | Altered | Case-control study | Mixed | MGS | [35] |
| **Mogibacterium** | Deficit-SCZ | ↑ | Case-control study | Mixed | 16S | [25] |
| **Mollicutes RF9** | SCZ | ↑ | Narrative Review | NA | NA | [4] |
| **Morganella** | SCZ-N | ↑ | Case-control study | Mixed | 16S | [59] |
| **Mycobacterium virus Renaud18 [virus]** | SCZ | ↑ | Case-control study | Treated | MGS | [16] |
| **Neisseria [genus]*** | SCZ | ↑ | Case-control study | Treated | 16S | [28] |
| **Odoribacter** | SSD<br>SCZ-N | ↓<br>↑ | Case-control study<br>Case-control study | Mixed<br>Mixed | MGS<br>16S | [20]<br>[59] |
| **Olsenella** | SCZ | ↑ | Case-control study | Treated | 16S | [29] |
| **Oscillospira** | SCZ | ↑ | Cohort Study | Treated | 16S | [26] |
| **Pachyviridae [virus]** | SCZ | Altered | Case-control study | Mixed | MGS | [35] |
| **Paraprevotella** | SCZ | ↓ | Case-control study | Treated | 16S | [29] |
| **Penicillium [fungi]** | FEP | ↓ | Case-control study | Drug naive | ITSs | [51] |
| **Peptococcus** | Deficit-SCZ | ↑ | Case-control study | Mixed | 16S | [25] |
| **Peptostreptococcaceae [family]** | SCZ<br>SCZ-N | ↑<br>↑ | Case-control study<br>Case-control study | Mixed<br>Mixed | MGS<br>16S | [35]<br>[59] |
| **Phascolarctobacterium** | SCZ | ↑ | Case-control study | Mixed | 16S | [67] |
| **Plasmodium relictum [fungi]** | SCZ | ↑ | Case-control study | Treated | MGS | [16] |
| **Porphyromonas [genus]*** | SCZ | ↑ | Case-control study | Treated | 16S | [28] |

| | | | | | | |
|---|---|---|---|---|---|---|
| **Prevotella** | SCZ<br>Deficit-SCZ | ↑<br>↑<br>↑ | Systematic review<br>Case-control study<br>Case-control study | NA<br>Mixed<br>Mixed | NA<br>16S<br>16S | [5]<br>[25]<br>[59] |
| **Prevotella copri** | SSD | ↓ | Case-control study | Mixed (mainly antipsychotics) | MGS | [20] |
| **Prevotellaceae [family]** | SSD | ↑ | Mendelian Randomization | NA | MR | [32] |
| **Prevotellaceae NK3B31 group** | Deficit-SCZ | ↑ | Case-control study | Mixed | 16S | [25] |
| **Proteobacteria [phylum]** | SCZ | ↑<br>↑ | Systematic review<br>Narrative Review | NA<br>NA | NA<br>NA | [5]<br>[7] |
| **Pseudobutyrivibrio xylanivorans** | SCZ | ↓ | Case-control study | Treated | MGS | [16] |
| **Purpureocillium [fungi]** | SSD | ↑ | Systematic review | NA | NA | [40] |
| **Raport Bacteroides–Prevotella ↑** | SCZ | ↑ | Case-control study | Mixed | MGS | [47] |
| **Rhizopus [fungi]** | FEP | ↓ | Case-control study | Drug naive | ITSs | [51] |
| **Rhodospirillaceae [family]** | SSD | ↓ | Mendelian Randomization | NA | MR | [32] |
| **Rikenellaceae** | SCZ | ↑ | Case-control study | Mixed | MGS | [35] |
| **Rikenellaceae RC9 gut group** | Deficit-SCZ | ↑ | Case-control study | Mixed | 16S | [25] |
| **Romboutsia** | SCZ-N | ↑ | Case-control study | Mixed | 16S | [59] |
| **Roseburia [genus]** | SCZ | ↓ | Systematic review<br>Narrative Review | NA<br>NA | NA<br>NA | [5]<br>[7] |
| **Roseburia [genus]** | SCZ | ↓ | Systematic review | NA | NA | [8] |
| **Roseburia hominis** | SCZ | ↓ | Case-control study | Treated | MGS | [16] |
| **Rothia*** | SCZ | ↓ | Cohort Study<br>Case-control study | Treated<br>Treated | 16S<br>16S | [26]<br>[28] |
| **Rountreeviridae [virus]** | SCZ | Altered | Case-control study | Mixed | MGS | [35] |

| | | | | | | |
|---|---|---|---|---|---|---|
| **Ruminiclostridium 5 [genus]** | SSD | ↑ | Mendelian Randomization | NA | MR | [32] |
| **Ruminococcaceae [family]** | SCZ | ↓ | Case-control study | Mixed | 16S | [59] |
| **Ruminococcaceae [family]** | SCZ | ↓ | Narrative Review | NA | NA | [71] |
| **Ruminococcus bicirculans** | SSD | ↓ | Case-control study | Treated | MGS | [9] |
| **Ruminococcus lactaris** | SSD | ↓ | Case-control study | Treated | MGS | [9] |
| **Ruminococcus torques** | FEP<br>Acute Phase | ↓<br>↑ | Case-control study<br>Case-control study | Drug naive<br>Mixed | Multi-omics<br>16S | [45]<br>[107] |
| **Ruthenibacterium lactatiformans** | SSD | ↑ | Case-control study | Mixed (mainly antipsychotics) | MGS | [20] |
| **Saccharomyces cerevisiae [fungi]** | SSD | ↓ | Narrative Review | NA | NA | [43] |
| **Saitozyma [fungi]** | FEP | ↑ | Case-control study | Drug naive | ITSs | [51] |
| **Schaalia*** | SCZ | ↓ | Case-control study | Treated | 16S | [28] |
| **Schitoviridae [virus]** | SCZ | Altered | Case-control study | Mixed | MGS | [35] |
| **Sellimonas intestinalis** | SSD | ↑ | Case-control study | Treated | MGS | [9] |
| **Shigella** | SCZ | ↑ | Case-control study | Treated | Multi-omics | [33] |
| **Siphoviridae** | SCZ | ↑ | Case-control study | Mixed | MGS | [52] |
| **Slackia** | Deficit-SCZ | ↑ | Case-control study | Mixed | 16S | [25] |
| **Streptococcus gallolyticus** | SCZ | ↑ | Case-control study | Treated | MGS | [16] |
| **Streptococcus gordonii** | SCZ | ↑ | Case-control study | Treated | MGS | [16] |
| **Streptococcus parasanguinis** | SSD | ↑ | Case-control study | Mixed (mainly antipsychotics) | MGS | [36] |
| **Streptococcus pasteurianus** | SSD | ↑ | Case-control study | Mixed (mainly antipsychotics) | MGS | [36] |
| **Streptococcus *salivarius*** | SCZ | ↑ | Narrative Review | NA | NA | [39] |
| **Streptococcus sobrinus** | Acute Phase | ↑ | Case-control study | Mixed | 16S | [107] |
| **Streptococcus vestibularis** | SCZ | ↑<br>behavior change in mice post FMT | Case-control study<br>Narrative Review | Treated<br>NA | MGS<br>NA | [16]<br>[39] |

| | | | | | | |
|---|---|---|---|---|---|---|
| **Subdoligranulum** | SCZ | ↓ | Case-control study | Treated | 16S | [30] |
| **Succinivibrio** | SCZ | ↑<br>↑<br>↑ | Systematic review<br>Meta-analysis<br>Narrative Review | NA<br>NA<br>NA | NA<br>NA<br>NA | [5]<br>[6]<br>[14] |
| **Suoliviridae [virus]** | SCZ | Altered | Case-control study | Mixed | MGS | [35] |
| **Sutterella** | SSD | ↓ | Case-control study | Mixed (mainly antipsychotics) | MGS | [20] |
| **Terrisporobacter [genus]** | SSD | ↓ | Mendelian Randomization | NA | MR | [32] |
| **Treponema*** | SCZ [elders] | ↑ | Case-control study | Treated (antipsychotics) | 16S | [38] |
| **Turicibacteraceae** | SCZ | ↑ | Case-control study | Mixed | MGS | [35] |
| **Veillonella** | SCZ | ↑ | Narrative Review | NA | NA | [39] |
| **Veillonella rogosae** | SCZ | ↓ | Case-control study | Treated | Shotgun | [9] |
| **Veillonellaceae** | SCZ | ↓ | Narrative Review | NA | NA | [48] |
| **Verrucomicrobia [phylum]** | SCZ | ↑ | Case-control study | Mixed | 16S | [11] |
